# 629. Hot Wheels- Burn Injuries from Lithium Battery-Powered Electric Bikes: Case Series from Urban Burn Center

**DOI:** 10.1093/jbcr/irag033.455

**Published:** 2026-04-06

**Authors:** Lara Bennett, Philip H Chang, Kathryn E Harris

**Affiliations:** William Randolph Hearst Burn Center, NY, New York; William Randolph Hearst Burn Center, NY, New York; William Randolph Hearst Burn Center, NY, New York

## Abstract

**Introduction:**

Lithium-battery powered electric bicycles are ubiquitous in the large urban centers of the United States, especially for food delivery and individual commuting. However, firefighters and burn centers have noted an increase in lithium-ion battery related burn injuries over the past few years with several of these patients requiring surgical treatment of full thickness burn injuries. Our burn team performed a 4-year retrospective examination of the burn patients treated at a major US urban burn center.

**Methods:**

A retrospective review was performed of all patients who sustained lithium battery associated burns from electric bikes from January 2022 through July 2025. Thirteen patients with burn wounds due to lithium battery fires were identified. Cases were reviewed for basic demographic information, burn depth, size, treatment, inhalation injuries, and hospitalization.

**Results:**

Patients in our case series were evenly divided with regards to gender (male 50%), mean age of 32 years (range 15-77 years), mean TBSA (24.6%). All but one patient had a combination of partial-thickness and full-thickness burns. All 12 patients required admission; 4 patients were intubated. One patient expired within 24 hours of admission per patient's previously stated advanced directives. Of the 12 cases, 10 required surgery with 3 patients requiring multiple surgeries.

**Conclusions:**

Our lithium ion burn patient population had a younger average age compared to our overall burn center population; inhalation injury was a significant factor in a number of these patients given that the ion battery fires led to apartment fires generating significant smoke and frequently trapping residents. Surgical grafting was required in the majority of these patients.

**Applicability of Research to Practice:**

As personal micromobility devices and electric-powered vehicles become more widespread throughout the U.S., burn centers across the country will see increasing numbers of burn patients injured by lithium ion battery explosions. Our shared experience can help other burn centers prepare to treat patients with these injuries and also provide information for firefighter advocacy groups to work towards improved legislative regulation of these batteries.

**Funding for the study:**

N/A.

